# Mobility and Fate of Cerium Dioxide, Zinc Oxide, and Copper Nanoparticles in Agricultural Soil at Sequential Wetting-Drying Cycles

**DOI:** 10.3390/ma12081270

**Published:** 2019-04-18

**Authors:** Mikhail Ermolin, Natalia Fedyunina, Olesya Katasonova

**Affiliations:** 1 Vernadsky Institute of Geochemistry and Analytical Chemistry, Russian Academy of Sciences, 19 Kosygin Street, Moscow 119991, Russia; katasonova_ol@mail.ru; 2 National University of Science and Technology “MISIS”, 4 Leninsky Prospect, 119049 Moscow, Russia; nataliafedyunina@mail.ru

**Keywords:** nanoparticles, cerium dioxide, zinc oxide, copper, soil, mobility, Wetting-Drying cycles

## Abstract

Study on the behavior and fate of nanofertilizers in soil plays a key role in the assessment of the efficiency of their use for intended purposes. The behavior of nanoparticles (NPs) in soil depends on environmental scenarios, such as Wetting-Drying cycles (WDCs). In the present work, the mobility and fate of CeO_2_, ZnO, and Cu NPs in agricultural soil at sequential WDCs have been studied. It has been shown that the mobility of CeO_2_ and ZnO NPs decreases after each WDC. After four WDCs the relative amount of CeO_2_ and ZnO NPs leached from soil decreases from 0.11 to 0.07% and from 0.21 to 0.07%, correspondingly. The decrease in the mobility of NPs is caused by their immobilization by water-stable soil aggregates, which are formed at sequential WDCs. Cu NPs are dissolved by soil solution, so their mobility (in ionic forms) increases after each subsequent WDCs. The relative content of Cu^2+^ sourced from Cu NPs increases up to 0.88% after four WDCs. It has been found that mineral NPs of soil can play an important role in the transport of insoluble engineered NPs. As for soluble NPs, the kinetics of their dissolution governs their mobility in ionic forms.

## 1. Introduction

The application of nanotechnologies to agriculture is regarded as promising approach to increasing crop production [1]. The use of so-called nanofertilizers and nanopesticides provides site-specific and controlled delivery of required ingredients (fertilizers and pest protectants) to plants [2,3,4]. Besides, an application of these “smart” nanoagrochemicals also enables their excess runoff to be significantly reduced. It is known, for example, that up to 70% of conventional agrochemicals remain unused [5], go to runoff, and can have adverse effects for ecosystem.

Engineered nanoparticles (NPs) of different chemical nature are proposed for use as nanofertilizers and nanopesticides [6]. For example, NPs of zeolite and hydroxyapatite (modified with urea or not) can be applied to the delivery of macronutrients (N, Ca, and P) to plants [7,8,9]. For the delivery of micronutrients, the application of metal and metal oxide NPs, such as CeO_2_ [10,11,12,13,14,15], ZnO [10,11,16,17,18,19,20], Zn [21], Cu [22], CuO [23], TiO_2_ [24,25], Au [26], etc. is also reported. Besides, metal and metal oxide NPs can also be used as nanopesticides, for example Ag [27], and Cu [28,29,30]. 

The study of the behavior and fate of nanofertilizers and nanopesticides in soil plays a key role in investigation of the efficiency of their use for intended purposes. It is known that NPs in soils can undergo various transformations, such as homo- and heteroaggregation, dissolution, chemical transformations, stabilization by organic matter, etc. [31,32], which affect mobility and bioavailability of NPs in soil. The study of mobility and speciation (forms of occurrence) of NPs in soil is very important in the assessment of their ability to reach plant rhizosphere for subsequent uptake. On the other hand, it should be noted that engineered NPs can be especially toxic [33,34], so NPs can spread in soil and negatively affect the living organisms. In this sense, NPs containing heavy metals (e.g. Ag, Cu, or Zn) attract a special attention as compared to mineral NPs (e.g. zeolite or hydroxyapatite).

The behavior of NPs in soils is a very complicated phenomenon [31], which depends on different parameters of soil, such as type of soil [35,36,37], clay content [38,39], content of organic matter [40,41], salinity [36,37,42], etc. It can be summarized that the mobility of NPs in soils decreases with increasing clay content, organic matter, and salinity of soil. However, the fate of NPs in soils is also dependent on environmental scenarios, such as Wetting-Drying cycles (WDCs). During WDCs most of the properties of soils can be severely affected [43,44,45], so the behavior of NPs in such soils may be complicated. Unfortunately, little is known about the behavior of NPs in soils exposed to sequential WDCs. For example, it is known that during WDCs capillary forces can govern attachment and detachment of NPs; besides, this process is size dependent [46]. It is also reported that capillary forces of colloids in films or air-water-solid interface are several orders of magnitude higher than Van der Waals attraction and electrostatic repulsion forces [47]. In addition, WDCs can change the ionic strength of soil solution significantly affecting the interaction between particles [48]. Therefore, the question of the effect of WDCs on the mobility of NPs is still open. The necessity to study the behavior of NPs in soil exposed to WDCs is also emphasized by Peijnenburg et al. [32]. It should be noted that this is extremely important for nanofertilizers and nanopesticides, which are exposed to sequential WDCs during irrigation of plants. At the present time, there is the knowledge gap regarding the effects of WDCs on fate of NPs in soil, which this work is intended to fill.

The aim of the present work is studying the mobility and fate of engineered NPs in soil at sequential WDCs. The study has been carried out using CeO_2_, ZnO, and Cu NPs, which are widely applied as potential nanofertilizers and nanopesticides, and a typical agricultural soil.

## 2. Materials and Methods 

### 2.1. Samples and Reagents

Nanoparticles of CeO_2_, ZnO, and Cu (Sigma Aldrich) with certified particle size distribution <50 nm, <100 nm, and 60-80 nm, correspondingly, were used. The study was performed using a sample of typical chernozem soil (Kursk region, Russia, 51°37′17.1″ N 36°15′42.0″E). The sample of the agricultural chernozem soil (horizon 0-10 cm) of arable land of conventional tillage was collected during fieldwork in June-July 2017 on the territory of the Kursk Research Institute of Agricultural Production. The sample was stored at room temperature and humidity. Prior to study, the soil sample was sieved through 1 mm screen.

Deionized water with resistivity 18.2 MΩ cm (Millipore, Moscow, Russia) was used at all stages of the research.

### 2.2. Characterization of NPs and Soil Sample

Size distributions of NPs under study were characterized by laser diffraction method (SALD-7500nano, Shimadzu, Moscow, Russia). The size and morphology of NPs were studied by scanning electron microscopy (JEOL JSM-6700F, JEOL, Moscow, Russia). The zeta-potential of NPs was measured using Malvern Zetasizer Nano ZS (Malvern Panalytical, Malvern, UK).

The elemental composition of soil sample before and after spiking with NPs was studied by inductively coupled plasma atomic emission spectrometry (ICP-AES, iCAP 6500 Duo, Thermo Scientific, Waltham, MA, USA) and mass spectrometry (ICP-MS, XSeries, Thermo Scientific, Waltham, MA, USA) after acid digestion. The digestion of soil (100 mg) was performed in an open beaker using a combination of three acids (HClO_4_, HF, HNO_3_). The digestion procedure was described in detail earlier [49]. Standard geological samples (Andesite, AGV-2, United States Geological Survey and Gabbro, GSO 521-84P, Russian Standard Sample) were used to control the completeness of digestion. The analysis was made in two replicates. According to the procedure used the relative standard deviation (RSD) for the determination of low concentrations of elements (less than 5×LOD, where LOD is the limit of detection) does not exceed 20%. For higher concentrations of elements (greater than 5×LOD), RSD does not exceed 10%.

The content of total organic carbon, nitrogen, and sulfur in soil was determined using CHNS analyzer (Vario EL III, Elementar, Langenselbold, Germany). Soil pH was measured in soil:water solution (1:2.5 wt.) by Hanna Instruments pH-meter.

### 2.3. Spiking Soil with NPs

NPs were added to soil samples as the suspensions: CeO_2_ and ZnO in water, while Cu in ethanol due to insufficient dispersibility in water. The suspensions were prepared from nanopowders using ultrasound treatment (60 W, Bandelin, Berlin, Germany). The ultrasonication time was pre-optimized (10 min). The amount of NPs in suspensions was chosen to reach the total concentrations of Ce, Zn, and Cu in spiked soil two times higher than in soil before spiking. The volume of the suspensions was chosen to reach complete wetting of soil sample (saturation of pore volume), but to avoid the formation of supernatant. The porosity of bulk soil sample calculated from its bulk density (~1.2 g cm^3^) was about 60%. The weight ratio of NP suspension to soil sample was 0.75 to 1. 

The suspensions (6 mL) of CeO_2_ (110 µg mL^−1^), ZnO (103 µg mL^−1^), and Cu (24 µg mL^−1^) NPs were carefully poured to three soil samples (8 g each) preliminarily placed in glass beakers. Then, the samples were left for 1 week for drying at room temperature and humidity. After drying, soil samples were gently crushed with a pestle to initial state and scrupulously mixed for homogenization, after that 2 g of spiked soils were taken for leaching experiments.

### 2.4. Performing Wetting-Drying Procedures

The procedure of spiking soil with NPs described above was regarded as 1st WDC. Each subsequent WDC was performed by adding water to soil samples at the ratio 0.75 to 1 (wt.) and drying for 1 week. Wetting soil sample spiked with Cu NPs was also made with water. After each WDC soil sample was gently crushed and homogenized; 2 g of soil was also taken at each WDC for leaching experiments. In total, four WDC were performed.

### 2.5. Leaching NPs from Soil Samples

**Figure 1:**
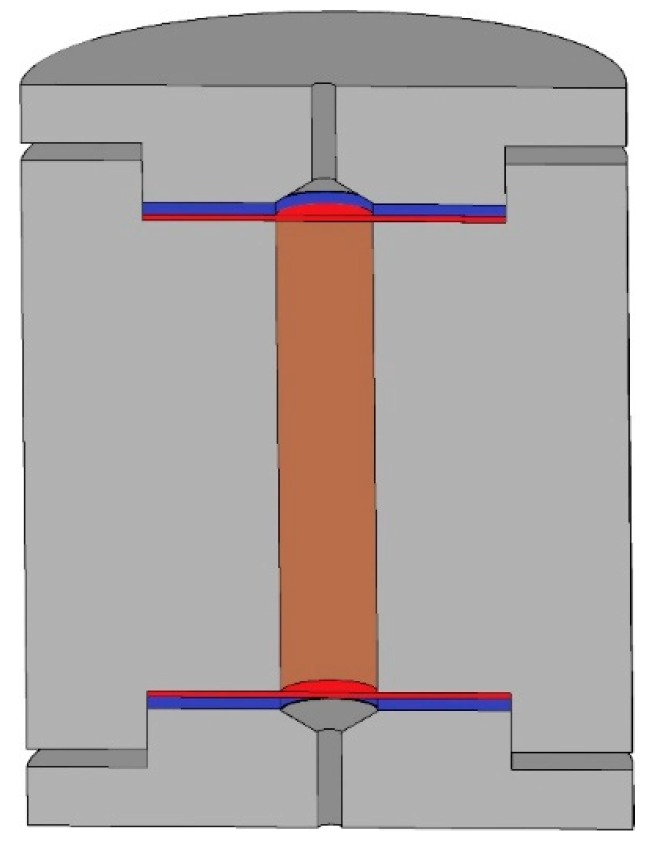
Cross-section of microcolumn: (gray); polytetrafluoroethylene (PTFE) column with screw caps, (blue); silicone spacers, (red)—membrane filters, (brown)—soil sample.

Leaching NPs from soil was performed using polytetrafluoroethylene (PTFE) microcolumn with following parameters: diameter - 7 mm, length - 28 mm, volume - 1 mL. The principal scheme of the column is presented in Figure 1. The leaching conditions were preliminarily optimized [50]. The optimization of conditions included the selection of eluent volume, which is sufficient for complete leaching of NPs from soil (see Supplementary Materials, Figure S1). Soil sample (0.2 g) was placed into the column, where it was held by cellulose membrane filters (Vladipor) with pore size 0.45 µm. The eluent (water) was pumped through the column at flow rate 1 mL min^−1^. The direction of flow was “from bottom to top” to achieve complete saturation of soil layer. The fractions of leachate (2 mL each) were collected at the outlet of the column for further analysis by ICP-MS. After leaching the residual fractions were removed from the column and analyzed by ICP-MS after digestion [49]. All the leaching experiments were made in triplicate.

### 2.6. Analysis of Leachates

The collected fractions of leachates were immediately analyzed by ICP-MS (Agilent 7900, Agilent, Santa Clara, CA, USA). The internal standard (Rh, 10 µg L^−1^) was used during the analysis. The concentrations of Ce, Zn, Cu in leachate fractions were determined at the following parameters: a RF generator power of 1550 W; a MicroMist nebulizer; a plasma-forming Ar flow rate of 15 L min^−1^; an Ar flow rate into the nebulizer of 1.05 L min^−1^; an analyzed sample flow rate of 1.0 mL min^−1^. The standard solutions (High-Purity Standards, USA) were used for calibration.

### 2.7. Control Experiments

The leaching experiments with soil without added NPs were used as control ones. Prior to leaching experiments, the initial soil samples were also exposed to four sequential WDCs as soil samples spiked with NPs. All the control experiments were made in triplicate. 

## 3. Results and Discussion

### 3.1. Characterization of NPs and Soil

**Figure 2:**
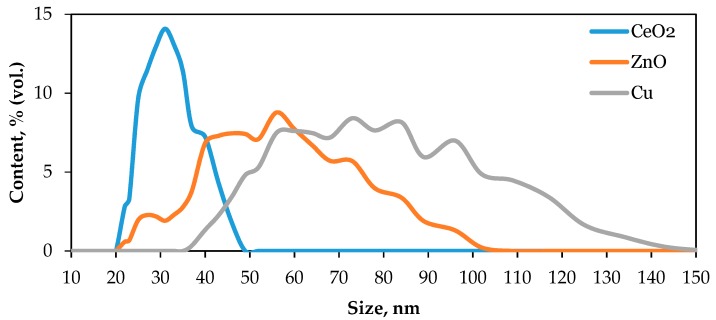
Size distributions of CeO_2_, ZnO, and Cu NPs in the suspensions prepared for spiking the soil.

Before commencing the study of WDC, the size distribution of CeO_2_, ZnO, and Cu NPs was characterized. The size distributions of NP suspensions prepared for spiking with soil are presented in Figure 2. The median diameters of CeO_2_, ZnO, and Cu NPs in the suspensions are 30, 50, and 70 nm, respectively. The measured zeta-potential of CeO_2_, ZnO, and Cu NPs is −24.2 ± 2.1, −25.7 ± 2.7, and −11.4 ± 1.8 mV, correspondingly. Low zeta-potentials (less than 30 mV in modulus) of NPs under study can cause their spontaneous homoaggregation with the lapse of time.

**Figure 3:**
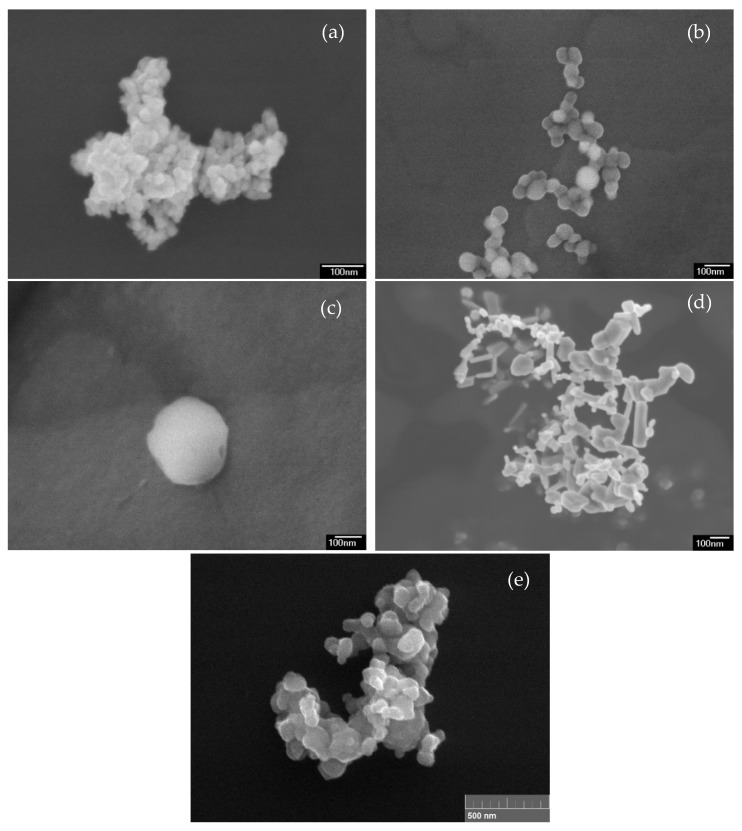
Micrographs of CeO_2_ (**a**–**c**), ZnO (**d**), and Cu (**e**) NPs.

The size and morphology of NPs were also studied by scanning electron microscopy; the micrographs are presented in Figure 3. In general, the size of CeO_2_ NPs (see Figure 3a) is in good agreement with obtained size distribution; however, particles up to about 300 nm are also presented (Figure 3b,c), but their amount is negligible. The sizes of ZnO (Figure 3d) and Cu (Figure 3e) NPs are in agreement with corresponding size distributions.

It has been shown that particle size distribution of prepared suspensions is in good agreement with certified values. This provides the reliability of the sample preparation step.

The soil is slightly alkaline (pH 7.80). The contents of organic carbon, nitrogen, and sulfur in soil are 29.3, 2.5, and 0.75 g kg^−1^, correspondingly. The concentrations of major elements as well as Ce, Zn, and Cu in soil sample before spiking are presented in Table 1. The concentrations of elements in standard samples are in good agreement with certified values; this confirm the reliability of the results of the analysis of soil.

NPs of CeO_2_, ZnO, and Cu were added to soil to reach the total concentration of Ce, Zn, and Cu in the spiked soil two times higher than in soil before spiking. The concentrations of Ce, Zn, and Cu in soil after spiking are 125 ± 4, 107 ± 3, and 39 ± 2 µg g^−1^, correspondingly. As is seen, the concentrations of Cu and Ce in the spiked soil correspond to expected ones (38 and 132 µg g^−1^, correspondingly. The concentration of Zn in the spiked soil (107 ± 2 µg g^−1^) is slightly lower than expected (120 µg g^−1^). This difference can be attributed to uneven distribution of NPs in soil.

### 3.2. The Mobility of CeO_2_, ZnO, and Cu NPs in Soil

**Figure 4:**
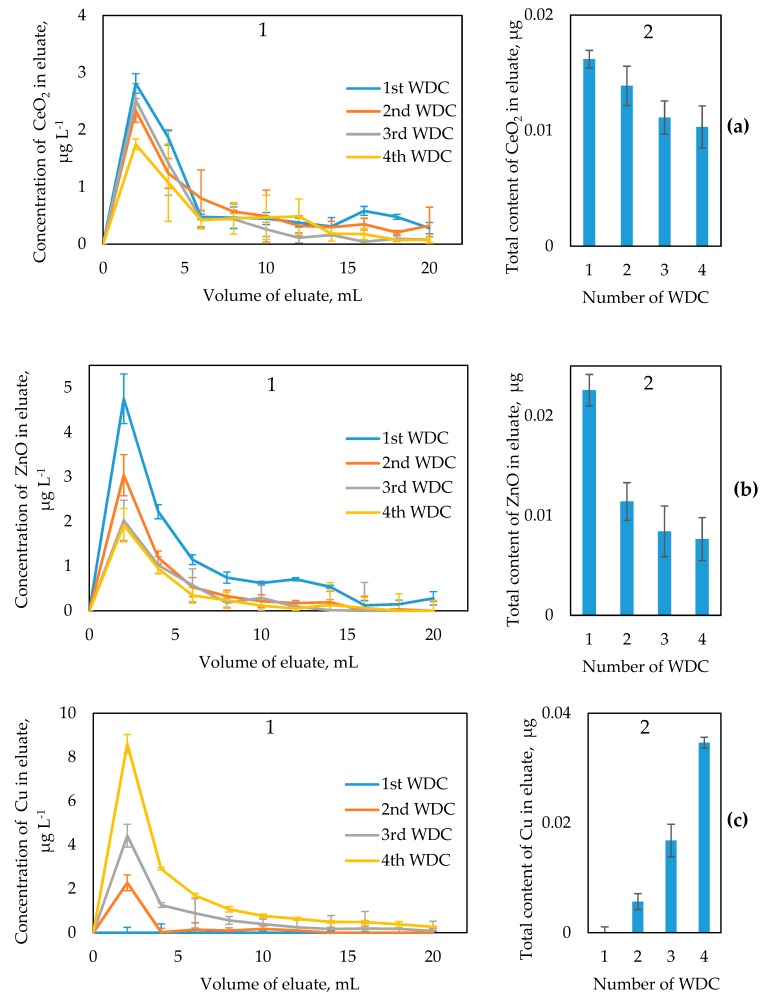
Mobility of CeO_2_ (**a**), ZnO (**b**), and Cu (**c**) NPs in soil depending on a number of WDC: (**1**) elution curves of NPs, (**2**) total content of NPs in leachates. Error bars indicate standard deviation.

On the basis of the results of analysis of leachates, the elution curves for CeO_2_, ZnO, and Cu NPs depending on a number of WDC were obtained (Figure 4). The concentrations of CeO_2_, ZnO, and Cu NPs in soil leachate were calculated after subtracting naturally occurring fraction (both particulate and truly dissolved, <0.45 µm) of corresponding elements determined in control experiments using soil without added NPs. The elution curves obtained in control experiments are presented in Supplementary Materials (Figure S2).

It has been shown that the mobility of all NPs under study in soil depends on a number of WDCs. The mobility of CeO_2_ NPs decreases after each subsequent WDC (Figure 4a). The maximum amount (0.016 µg) of leached CeO_2_ NPs was observed after 1st WDC. After 4th WDC, the amount of leached CeO_2_ NPs decreases down to 0.10 µg. In relative terms, the mobile fraction of CeO_2_ NPs decreases after four WDCs from 0.11 to 0.07% of the total content of CeO_2_ NPs added to soil. The decrease in the mobility of CeO_2_ NPs can be explained by aggregation processes in soil caused by sequential WDCs. It has been reported that WDCs induce the formation of stable in water aggregates in tilled soils [51]. Evidently, CeO_2_ NPs are also involved in this process, so their immobilization by soil aggregates occurs. In addition, taking into account low zeta-potential of CeO_2_ NPs, homoaggregation process can also cause the decrease in their mobility.

**Figure 5:**
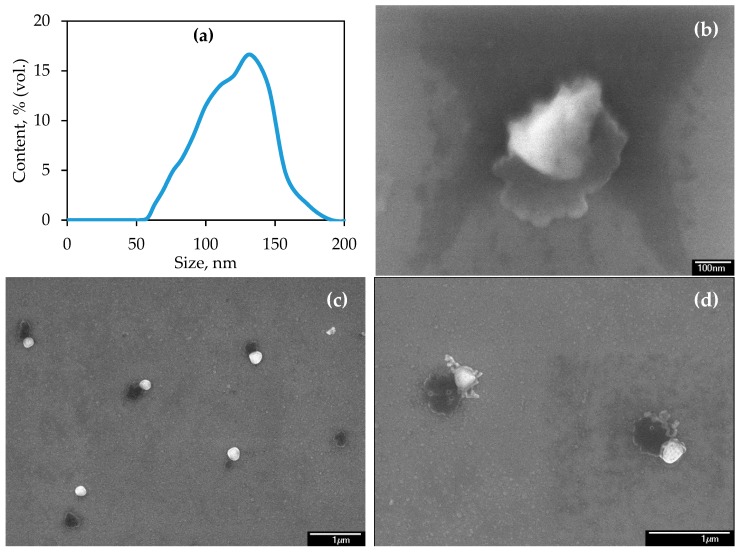
Size distribution of particles in the soil leachate (**a**). Micrographs of aggregates of CeO_2_ (brighter) and mineral (darker) NPs (**b**), their “tandems” (**c**), and “ensembles” (**d**) in the soil leachate.

It is known that CeO_2_ NPs are poorly soluble in water, so their fate in soil primarily depends on the homo- and heteroaggregation processes as well as interaction with soil organic matter. Therefore, CeO_2_ NPs can be leached from soil as individual particles and homo- or heteroaggregates (stabilized or not by soil organic matter). The micrographs and particle size distribution of soil leachate are presented in Figure 5. As is seen, soil leachate has size distribution <200 nm with median diameter 110 nm. Micrographs show that leachate contains mineral soil particles, CeO_2_ NPs, and their homo- and heteroaggregates. As is seen, CeO_2_ NPs are primarily leached from soil as heteroaggregates (Figure 5b). It has been also observed that larger CeO_2_ NPs present in leachate as individual particles as well as form “tandems” with mineral NPs of soil (Figure 5c). The presence of “ensembles” of CeO_2_ NPs of different size with mineral NPs is also seen (Figure 5d). It should be noted that heteroaggregates could be formed during sample preparation step for study by electron microscopy (drying the droplet of leachate).

It has been shown that mineral NPs of soil play an important role in transport of CeO_2_ NPs. Mineral NPs (mainly clay minerals) can serve as a carrier for engineered NPs in soils. Therefore, the mobility of CeO_2_ NPs in soil may depend on the amount of mineral NPs.

**Figure 6:**
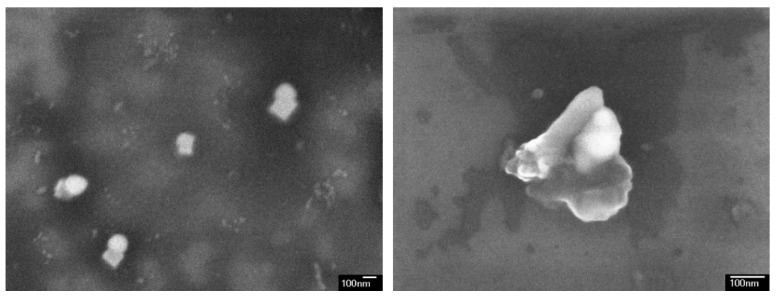
Micrographs of homo- (**a**) and heteroaggregates (**b**) of ZnO NPs in the soil leachate.

The similar behavior was observed for ZnO NPs, their mobility also decreased after each subsequent WDC (Figure 4b). The amount of leached ZnO NPs decreases from 0.023 to 0.008 µg after four WDCs. In relative terms, the mobile fraction of ZnO NPs decreases after four WDCs from 0.21 to 0.07% of the total content of ZnO NPs added to soil. The micrographs show that ZnO NPs are contained in leachate as homo- and heteroaggregates (Figure 6). Therefore, the mobility of ZnO NPs in the soil at sequential WDCs primarily depends on aggregation processes (as for CeO_2_ NPs).

It is known that NPs can dissolve in water (Wagner et al. 2014). The dissolution of NPs is controlled by a number of particle- and solution-specific properties: (1) the solubility constant, (2) chemical speciation (e.g. acid–base reactions, complexation, re-precipitation), and (3) the specific surface area and mass-transfer kinetics [52]. The dissolution of ZnO NPs can occur according to the reaction [53]:

ZnO (NPs) + H_2_O ↔ Zn^2+^ + 2OH^−^

Therefore, the fate of ZnO NPs can also be dependent on their dissolution. However, if the dissolution of ZnO NPs took place, there would be an increase of Zn concentration in leachate. In contrast, in present study the decrease of Zn concentration in leachate was observed.

The fate of Cu NPs is completely opposite to this of CeO_2_ and ZnO NPs. The mobility of Cu NPs increases after each subsequent WDC (Figure 4c). The study of leachate by electron microscopy did not show the presence of Cu NPs. Therefore, such behavior of Cu NPs is attributed to their dissolution during wetting of soil. Dissolution of zero-valent metals requires an oxidation step by dissolved oxygen. The dissolution of Cu NPs can occur according to the reaction [53]:

Cu (NPs) + 2H_2_O + O_2_ ↔ Cu^2+^ 4OH^−^

The total content of Cu^2+^ sourced from Cu NPs increased up to 0.035 µg after four WDCs (0.88% of the total Cu NPs in soil). It should be noted that after 1st WDC the leaching of Cu^2+^ derived from NPs was not observed. This can be attributed to the fact that 1st WDC was performed with ethanol (i.e. spiking soil with Cu NPs), where dissolution of Cu NPs was apparently impossible. Thus, each subsequent WDC leads to gradual dissolution of Cu NPs. The dissolution of Cu NPs was also observed in natural waters [54]. 

The dissolution rate plays an important role in NPs dissolution. Since the dissolution process is halted under dry condition, it depends largely on the ability of soil to retain moisture in macro- and micropores. In this sense, the dissolution of NPs should be lower in topsoil, which dries faster than deeper soil horizons. In present work, the averaged (over the four WDCs) dissolution rate of Cu NPs is about 0.009 µg/WDC. It should be noted that the Wetting-Drying conditions of soil samples under study more correspond to the ones, which may take place in topsoil. Therefore, the dissolution of Cu NPs in deeper soil horizons may be more intensive.

It should be noted that after 1st WDC the dissolution of Cu NPs was not observed. This can be explained by the fact that Cu NPs were added to soil as a suspension in ethanol, where dissolution is impossible. Each subsequent WDC was performed using water, so the gradual dissolution was observed.

In general, it has been shown that Cu NPs are completely retained in soil and gradually dissolved after each subsequent WDC. Sequential WDCs enable the dissolution of these Cu NPs to be controlled. This is interesting from the agricultural point of view and can be useful, for example, in application of Cu NPs as nanofertilizers in greenhouses for controlled release of nutrient ingredients (Cu^2+^) into soil by controlled irrigation.

### 3.3. Mass Balance of Ce, Zn, and Cu in Soil Fractions

The concentrations of elements in leachates as well as residual fractions were determined (Table 2). The concentrations of Ce, Zn, and Cu derived from NPs were also different from their “naturally occurring” concentrations in soil, obtained from the control experiments. It is shown that minor portion of Ce, Zn, and Cu is leached from soil (less than 1 µg g^−1^). The sum of fractions of elements under study is in good agreement with their total concentration in soil spiked with NPs; the estimated recovery of metals is close to 100%.

## 4. Conclusions

The behavior of NPs in soils is a very complicated phenomenon, which is dependent on different physical and chemical properties of soil. The present work has shown that fate and mobility of NPs in soils are also dependent on environmental scenarios such as number of WDCs. The fate and mobility of CeO_2_, ZnO, and Cu NPs in agricultural soil were investigated. It has been shown that the mobility of CeO_2_ and ZnO NPs decreases after each subsequent WDC. These NPs are insoluble and leached from the soil primarily as homo- and heteroaggregates. The decrease in the mobility of NPs is related to the formation of water-stable soil aggregates during WDCs. Therefore, CeO_2_ and ZnO NPs are also involved in this process, and their immobilization by soil aggregates occurs. On the contrary, Cu NPs dissolve in the soil solution, so their mobility (in the form of Cu^2+^) increases after each subsequent WDC.

In general, it can be concluded that mineral NPs of soil play an important role in the transport of insoluble engineered NPs. Evidently, mineral NPs can serve as a carrier for engineered NPs. Therefore, the mobility of NPs in soil may be dependent on the amount of mineral NPs. This conjecture needs further study.

Engineered NPs, which are soluble in water, can be dissolved in soil at sequential WDCs. The kinetics of NPs dissolution governs their mobility in ionic forms. Therefore, sequential WDCs enable the dissolution of these NPs to be controlled. This is interesting from the agricultural point of view and can be useful, for example, for the controlled release of active ingredients into soil from NPs by a number of WDCs (e.g. irrigation of plants in greenhouses).

## Figures and Tables

**Table 1:** Chemical composition of soil sample (*mean ± standard deviation*).

Element Oxide/ Element	Soil	Standard Sample			
		Andesite, AGV-2		Gabbro, GSO 521-84P	
		Determined	Certified Value	Determined	Certified Value
	*Concentration,* mg g^−1^				
Na_2_O	8.2 ± 0.3	41	41.9 ± 1.3	27	27.2 ± 0.7
MgO	10.5 ± 0.1	18	17.9 ± 0.3	67	68.1 ± 0.9
Al_2_O_3_	103 ± 2	168	169.1 ± 2.1	149	149.3 ± 1.6
P_2_O_5_	1.69 ± 0.01	4.6	4.8 ± 0.2	11	10.3 ± 0.2
K_2_O	22.5 ± 0.7	28	28.8 ± 1.1	31	30.9 ± 1.1
CaO	16.4 ± 0.7	50	52.0 ± 1.3	108	106.8 ± 1.4
TiO_2_	5.76 ± 0.02	10.6	10.5 ± 2.2	17	17.2 ± 0.5
MnO	0.84 ± 0.02	1.0	1.0 ± 0.03	1.8	1.7 ± 0.1
Fe_2_O_3_	37.1 ± 0.3	66	66.9 ± 1.3	112	113 ± 1
	*Concentration,* µg g^−1^				
Ce	66 ± 1	51.5	53 ± 4	53.4	58 ± 5
Zn	60 ± 2	93.4	86 ± 8	120	120 ±15
Cu	19 ± 1	67.2	68 ± 3	180	163 ± 20

**Table 2:** Concentration of Ce, Zn, and Cu in soil fractions as obtained by inductively coupled plasma mass spectrometry (ICP-MS) (*mean ± standard deviation*).

Element	Number of WDC	Concentration, µg g^−1^		
		NPs-Derived Fraction	Naturally Occurring Fraction	Residual Fraction
Ce	1	0.073 ± 0.004	0.007 ± 0.001	123 ± 3
	2	0.066 ± 0.008	0.009 ± 0.001	
	3	0.053 ± 0.007	0.011 ± 0.001	
	4	0.049 ±0.009	0.011 ± 0.003	
Zn	1	0.091 ± 0.006	0.088 ± 0.006	108 ± 4
	2	0.046 ± 0.008	0.084 ± 0.007	
	3	0.034 ± 0.010	0.087 ± 0.010	
	4	0.033 ± 0.009	0.075 ± 0.009	
Cu	1	-	0.133 ± 0.005	35 ± 3
	2	0.028 ± 0.007	0.136 ± 0.007	
	3	0.084 ± 0.015	0.147 ± 0.015	
	4	0.173 ± 0.005	0.139 ± 0.005	

## Supplementary Materials

The following are available online at https://www.mdpi.com/1996-1944/12/8/1270/s1, Figure S1: Elution curve of CeO_2_ NPs from soil, Figure S2: Elution curves of Ce, Zn, and Cu for soil before spiking with NPs depending on a number of WDCs.
