# Supplementary material for: Mobility and Fate of Cerium Dioxide, Zinc Oxide, and Copper Nanoparticles in Agricultural Soil at Sequential Wetting-Drying Cycles
Source: Materials (Basel). 2019 Apr 18;12(8):1270. doi: 10.3390/ma12081270 (PMC6514903; doi:10.3390/ma12081270)
Supplement: Supplementary file 1 [file materials-12-01270-s001.pdf]

materials

Supplementary Information

Mobility and Fate of Cerium Dioxide, Zinc Oxide, and Copper Nanoparticles in Agricultural Soil at Sequential Wetting–Drying Cycles

Mikhail Ermolin [1,*], Natalia Fedyunina [2] and Olesya Katasonova [1]

[1] Vernadsky Institute of Geochemistry and Analytical Chemistry, Russian Academy of Sciences, 19 Kosygin Street, Moscow 119991, Russia; katasonova_ol@mail.ru (O.K.)
[2] National University of Science and Technology "MISIS", 4 Leninsky Prospect, 119049 Moscow, Russia; nataliafedyunina@mail.ru (N.F.)
* Correspondence: ermolin@geokhi.ru (M.E.); Tel.: +7-499-939-7838

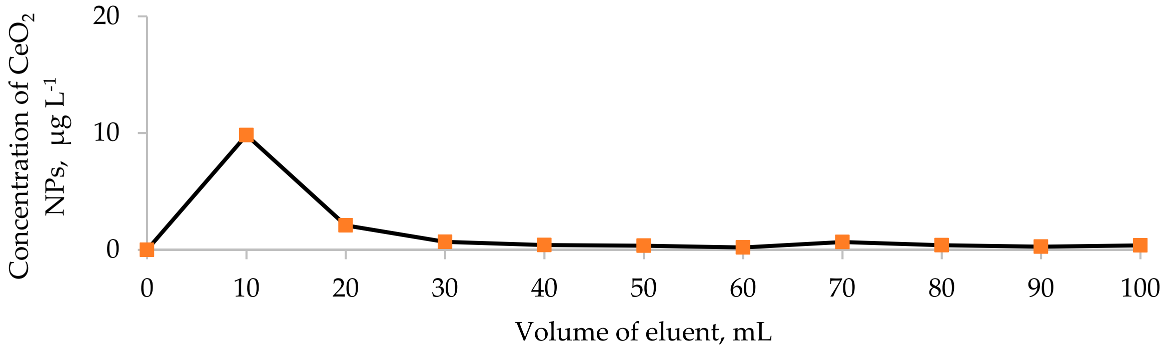

Figure 1. Elution curve of CeO_2 NPs from soil.

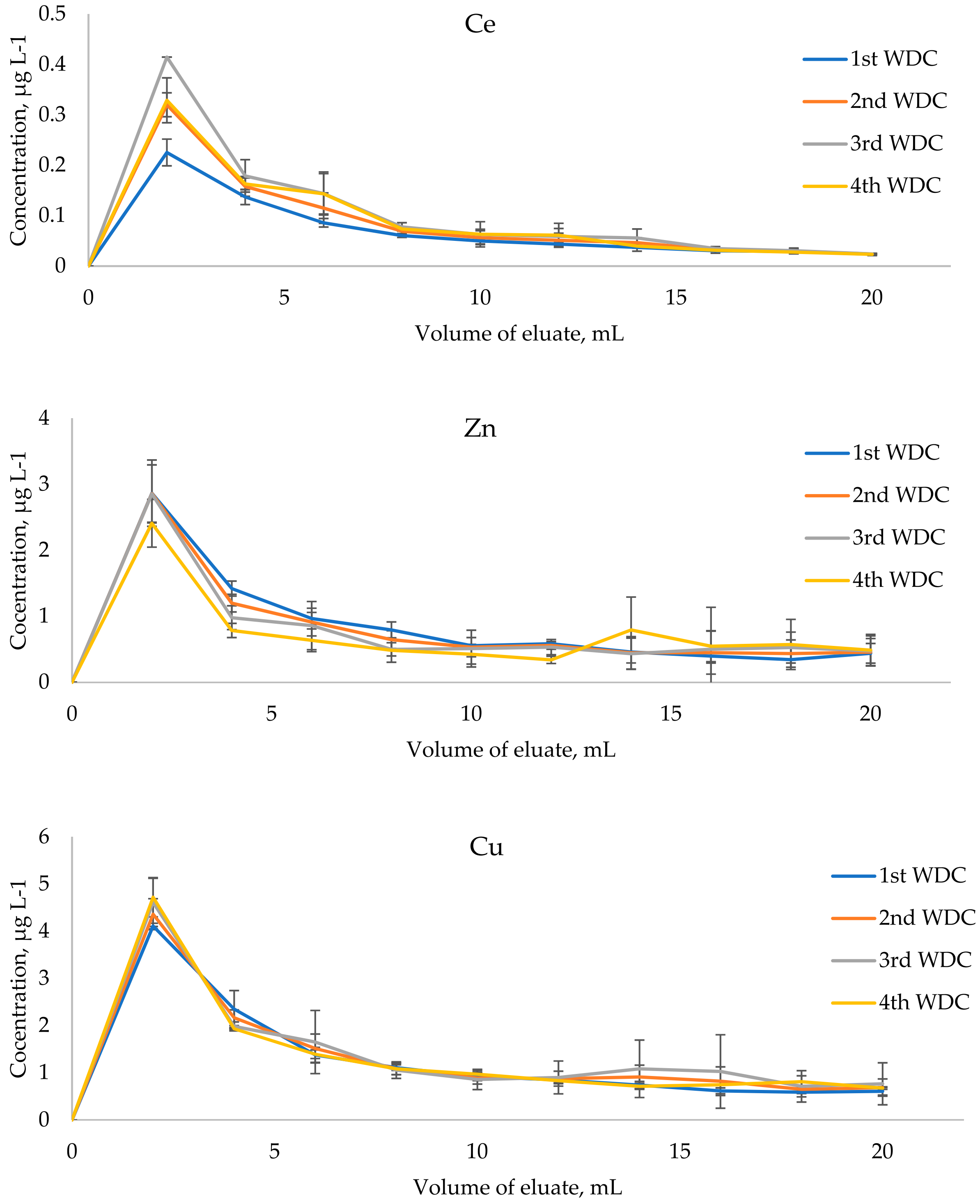

Figure 2. Elution curves of Ce, Zn, and Cu for soil before spiking with NPs depending on a number of WDCs.
